# The Epidemiology of Young People’s Work and Experiences of Violence in Nine Countries: Evidence from the Violence against Children Surveys

**DOI:** 10.3390/ijerph192416936

**Published:** 2022-12-16

**Authors:** Amiya Bhatia, Maryam Parvez, Jodie Pearlman, Fred Kasalirwe, Ligia Kiss, Agnes Kyamulabi, Eddy J. Walakira, Karen Devries, Clare Tanton

**Affiliations:** 1 Department of Global Health and Development, London School of Hygiene and Tropical Medicine, London WC1E 7HT, UK; 2 Department of Epidemiology, Biostatistics and Occupational Health, School of Population and Global Health, McGill University, Montréal, QC H3A 1G1, Canada; 3 School of Economics, Makerere University, Kampala P.O. Box 7062, Uganda; 4 Institute for Global Health, University College London, London WC1E 6BT, UK; 5 Department of Social Work and Social Administration, Makerere University, Kampala P.O. Box 7062, Uganda

**Keywords:** work, violence, young people, child labour, survey, epidemiology

## Abstract

Globally, 497 million young people (15–24 years) are in the labour force. The current research on work and violence indicates reciprocal links across the life course. This study draws on data from 35,723 young people aged 13–24 years in the Violence Against Children Surveys (VACS) in nine countries to describe the epidemiology of work in order to explore associations between (1) current work and violence and (2) childhood violence and work in a hazardous site in young adulthood. The prevalence of past-year work among 13–24-year-olds was highest in Malawi: 82.4% among young men and 79.7% among young women. In most countries, young women were more likely to be working in family or domestic dwellings (range: 23.5–60.6%) compared to men (range: 8.0–39.0%), while men were more likely to be working on a farm. Work in a hazardous site was higher among young men compared to women in every country. Among children aged 13–17 years, we found significant positive associations between past-year work and violence among girls in three countries (aORs between 2.14 and 3.07) and boys in five countries (aORs 1.52 to 3.06). Among young people aged 18–24 years, we found significant positive associations among young women in five countries (aORs 1.46 to 2.61) and among young men in one country (aOR 2.62). Associations between childhood violence and past-year work in a hazardous site among 18–24-year-olds were significant in one country among girls and in three countries among boys. Continued efforts are needed to prevent hazardous work, improve work environments, and integrate violence prevention efforts into workplaces.

## 1. Introduction

Globally, 497 million young people aged 15–24 years are in the labour force, either employed or unemployed [1]. Among children, the International Labour Organisation (ILO) reports that over 222 million children aged 5–17 years are in employment [2], and 160 million children were engaged in child labour in 2020, of which 79 million were engaged in hazardous work [2]. Children’s work can include household chores, agriculture, and economic activities, or it can take more hazardous forms (e.g., mining, construction, and factory work). This work can be unpaid or paid through a wage, salary, cash, or in-kind contribution [1]. While governments and institutions have made efforts to increase access to the labour market, reduce unemployment, invest in skills training, and improve work environments for young people, global estimates suggest that young people are three times as likely as adults (25 years and older) to be unemployed. ILO estimates an unemployment rate of 13.6% with large variation by region, and higher rates of unemployment among young women compared to men [1]. Among those working, many young people work in precarious conditions and continue to experience extreme poverty, and close to 96% of young people who are working in South Asia and sub-Saharan Africa are in informal sectors or employment [1].

International and national laws have sought to govern and regulate work: Article 32 of The UN Convention on the Rights of the Child calls on state parties to implement a minimum age for employment and regulate hours and working conditions to protect children from economic exploitation [3]. Both the ILO Minimum Age Convention (1973) [4] and the Worst Forms of Child Labour Convention [5] provide further guidance on the types and hours of work children should participate in. Global and national human rights organisations have also made efforts to monitor hazardous child labour, improve government accountability [6,7,8,9], and initiate youth-led campaigns to eradicate child labour [10].

Although public health research has examined the adverse effects of work and hazardous work on health outcomes for over a century, more limited attention has been given to how children and young people’s work affects their health and to how work affects violence. Although global estimates suggest one billion children aged 2–17 years experienced past-year violence [11], including at least one in two children in Asia, Africa, and North America, less is known about employer violence, the violence children experience in and around workplaces, and about the violence they may experience for either working or not working. Currently, most of the evidence on links between work and violence centres on adult women’s work and experiences of violence, particularly intimate partner violence [12,13,14,15,16,17]. In 2006, the UN Secretary-General’s Study on Violence against Children described how forms of child labour and hazardous work increased the risk of violence and also highlighted the invisibility of prevalence estimates for violence experienced by children in the workplace [18].

Existing research finds reciprocal links between work and violence across the life course. Three primary findings emerge: that work can either (1) increase or (2) decrease the risk of violence, and that (3) childhood violence shapes work trajectories, affecting work outcomes later in life. First, evidence suggests that engaging in work is associated with an increase in violence for children and young people. Country- and context-specific studies have highlighted the extent of workplace violence perpetrated by employers or customers. For example, a study in Afghanistan found that, after the home, the workplace was the second most predominant place of occurrence of violence against children [19]. A study in Turkey found that the majority of children working full-time at a workplace experienced violence [20]. In a study in Uganda, two in five young people had experienced workplace violence in the past year [21]. In particular, child labour increases the risk of experiencing violence and exploitation [22,23,24], alongside adverse health [25] and education outcomes [26].

A second finding in the current scholarship on young people’s work and violence suggests that engaging in work and having an income can prevent violence, particularly among women. Findings from a cohort study among adult women in South Africa showed that paid work was associated with a reduction in IPV [14]. A study in Malawi found that unemployment was associated with experiences of severe IPV, particularly among youth aged 15–24 years [16].

Thirdly, there is evidence from longitudinal studies that experiencing violence or neglect in childhood is associated with adverse and unequal work outcomes (income, employment, job skill, assets, financial insecurity) later in life [27]. Cohort studies in the USA [28] and Norway [29] found that children who experienced violence had lower levels of employment and earnings in adulthood compared to those who did not experience violence. Of the limited research in low- and middle-income countries (LMICs), a longitudinal study in Cape Town found that any experience of physical or emotional abuse during childhood was associated with a later 12% loss of wages, with larger wage losses among young women compared to men [30]. There is also evidence to suggest that childhood violence leads to poorer educational outcomes [31], which affects both access to work and wages [32].

Key gaps in the literature remain. There is limited evidence from LMICs, including few comparative and cross-country studies on the epidemiology of work among children and young people and how experiences of violence vary by employment status. Most studies among children examine violence perpetrated by employers or in workplaces, with more limited attention to how working could change children’s exposure to violence both within and beyond the workplace, including at home, in public spaces, or at school. There is also limited evidence on whether childhood violence is associated with being in hazardous or harmful work in young adulthood in LMICs.

Our study uses the Violence Against Children Surveys (VACS), national surveys conducted in over 20 LMICs with young people aged 13–24 years. VACS include data on lifetime, childhood, and past-year violence, alongside data on young people’s experiences of work and hazardous work in the past year. Only a small number of studies have examined work in the VACS: one study using data from Haiti found an increased risk of physical, sexual, or emotional violence before the age of 18 among child domestic servants compared to other children [23]. Another study using data from Nigeria found that young people of the highest economic status were more likely to report experience of violence in the past year than those of the lowest economic status [33].

This study is informed by the multiple and reciprocal links between work and violence, current gaps in the literature, and the opportunities VACS offer to study work and violence. First, we describe the prevalence of work and hazardous work for young women and men aged 13–24 years in nine countries and explore how the epidemiology of work varies by country context and sex. We hypothesise that there will be both across-country and within-country variation in the prevalence of work and violence disaggregated by age and sex. We then examine associations between current work and violence among children (13–17 years) and young adults (18–24 years). We hypothesise that children and young people who are working will have higher odds of violence compared to children and young people who are not working. Finally, we examine associations between childhood violence (occurring at or before 18 years of age) and hazardous labour among young people aged 18–24 years. We hypothesise that the early experiences of violence increase the odds of experiences of hazardous labour among young people.

We theorise work and violence as structurally produced, gendered, and unequally distributed among children and young people. Forms of hazardous work, in particular, are products of economic and social inequalities which adversely affect children. Given the gendered nature of work and the ways in which the types of work and policy frameworks for work change as children become young adults, we draw on a life-course and gender-responsive approach to stratify by sex and age.

## 2. Methods

### 2.1. Data Sources

The VACS use a three-stage cluster and split-sample design to select only one respondent aged 13–24 years per household. To reduce the likelihood that survivors and perpetrators of violence are interviewed in the same household, women and men are sampled separately and are interviewed in different primary sampling units or enumeration areas [34]. Young people are interviewed by trained enumerators about lifetime and past-year violence, work, school, sexual health, mental health, substance use, and social support. A separate survey is used to collect household-level socioeconomic information. Informed consent or assent is obtained from all participants prior to enrolment in the survey, considering the rights of parents or guardians to make an informed decision about their child’s participation. The VACS adhered to guidelines for conducting research with children [35], and the interviewers were trained to provide referrals to violence and social services [36].

We considered surveys that were publicly available on the Together for Girls website in June 2021 [37] and received additional government approval for the Uganda survey. We included surveys with some comparable data on past-year work and violence. Out of these 12 surveys, 9 surveys met our criteria, and we excluded 3 surveys: Cambodia, 2013 [38], as no data were collected on past-year work; Haiti, 2012 [39]; and Tanzania, 2009 [40], as categorisations of violence and work differed substantially from other countries and were not comparable.

### 2.2. Measures of Young People’s Work

Past-year work was defined as any work, paid or unpaid, in the past 12 months in eight out of nine surveys. In the Kenya survey, participants were asked specifically about paid work: “in the last 12 months, how often have you worked for money or any other payment/goods?” We defined a binary variable for past-year work with the denominator as all young people who participated in the survey.

In seven out of nine surveys, the participants were asked similar questions about their primary worksite in the past year. To examine the location of work, we grouped worksites into five categories: family dwelling, farm/garden, shop/restaurant (including kiosk, restaurant, hotel, café, bar, fixed, street, or market stall), hazardous worksite (factory/workshop, construction site, or mine/quarry), and other (followed by a write-in option). In the Colombia survey, data on worksites were not comparable. In the Kenya survey, participants reported separately on five forms of hazardous work (working in a mine; over open flames; with sharp tools, such as a machete; with heavy equipment; and in a factory). We defined two binary variables for past-year hazardous work to (1) estimate the prevalence of hazardous work among all young people who participated in the survey and (2) estimate the proportion of work that was hazardous among participants who were working. The Kenya survey also asked participants to report how many hours they worked on average per day in the past 12 months. Based on ILO guidance, we created an additional binary variable for this survey (working less than 9 h or 9 h or more [41]).

Finally, we examined decision-making about money earned. In six surveys (Colombia, El Salvador, Honduras, Kenya, Uganda, and Zimbabwe), participants who worked were asked the following question: “who usually decides how the money you earned will be used?” We defined a categorical variable for decision-making about money earned (participant decides, joint decision with someone else, decision by a partner (husband or boyfriend), and decision by someone else/other). Supplementary Table S1 summarises the measures of work in each country.

### 2.3. Measures of Young People’s Experiences of Violence

Physical violence was measured with three items in six of the nine countries (Malawi, Nigeria, Zambia, Zimbabwe, Kenya, and Uganda): (1) punching, kicking, whipping, or beating with an object; (2) choking, smothering, drowning, or burning; and (3) using or threatening with a knife, gun, or other weapon. In three countries (El Salvador, Colombia, and Honduras), there was an additional item to measure physical violence: slapping, pushing, shoving, shaking, or intentionally throwing something to hurt. Physical-violence questions were asked for each of the following perpetrators: partners, peers, caregiver/relatives, or other adult (teacher, religious or community leader, and neighbours). In the Kenya survey, peers were not included in the measure of physical violence, and “other adult” referred specifically to public authority figures.

Sexual violence was measured consistently across all countries and defined as any of four acts: (1) touched you in a sexual way without your permission but did not try to force you to have sex, (2) tried to make you have sex against your will but did not succeed, (3) physically forced you to have sex and did succeed, and (4) pressured you to have sex and did succeed.

Emotional violence was measured by using three or four variables in each country. Across all countries except Kenya, it was defined as any of three acts by a caregiver/relative: (1) told you that you were not loved or did not deserve to be loved; (2) said they wished you had never been born or were dead; and (3) ridiculed you or put you down by, for example, saying that you were stupid or useless. Colombia and El Salvador included an additional act of (4) threatened to get rid of you. In two countries, emotional violence by a peer was also measured. In Colombia, this was defined as being bullied at least once in the past 30 days. In Zambia, this was measured by using three acts: (1) made you get scared or feel really bad because they were calling you names, saying mean things to you, or saying they did not want you around; (2) told lies or spread rumours about you or tried to make others dislike you; and (3) kept you out of things on purpose, excluded you from their group of friends, or completely ignored you. In Kenya, emotional violence was defined as ever experiencing any of four acts by anyone: (1) said or did something on purpose to humiliate you in front of others, (2) made you feel unwanted, (3) threatened to abandon you, and (4) abandoned you.

We defined childhood violence as any form of physical or sexual violence before age 18 years and past-year violence as any experience of physical or sexual violence in the past 12 months. For childhood and past-year violence, we constructed binary variables: the numerator was participants who reported violence, and the denominator included participants who answered no to all questions about physical, sexual, or emotional violence in the time period [42], or who reported that they do not know or did not answer the question. Because forms of violence are interconnected, only participants who had never experienced violence in a specified time period were in the denominator. We did not include emotional violence in the numerator due to the varying severity of forms of emotional violence captured in questions asked across surveys.

### 2.4. Covariates

We included the covariates related to violence and work. Age was included as a continuous variable. The following covariates were included as binary variables: orphan status (ref = one or both parents alive), child marriage (ref = marriage after age 18 years or currently unmarried), currently married (ref = unmarried), sex of the head of household (ref = female), type of toilet facility as a proxy for socioeconomic status (ref = no flush toilet), and ever attended school (ref = no).

### 2.5. Analysis

Our first aim was to describe the prevalence of work among young people aged 13–24 years. We estimated the prevalence of paid or unpaid work and of work in a hazardous site in the past year stratified by sex in each country. We then additionally stratified by age to examine the prevalence of work among children (13–17 years) and young people (18–24 years), and, among participants who reported work, we calculated the proportion working in a hazardous site. Finally, we described worksites and who decided how money earned from work was spent by sex in each country. If participants selected “other” for worksite, we summarised open text responses, using word clouds to explore which worksites did not fit into existing response options.

Our second aim was to examine the experiences of past-year physical and/or sexual violence among participants who were working in the past year compared to those who were not working. We conducted descriptive analyses stratified by age (13–17 years compared to 18–24 years) and sex (girls compared to boys). Chi-square tests and corresponding *p*-values were used to assess differences in the prevalence of violence by work status. We then specified null and adjusted generalised linear models (GLMs) with a binomial link function with past-year physical and/or sexual violence as the outcome and past-year work as the exposure.

For our third aim, we sought to examine associations between childhood physical and/or sexual violence and hazardous worksites in young adulthood. We restricted our analysis to young men and women aged 18–24 years so that violence exposures were measured before work outcomes. We described the prevalence of childhood physical and/or sexual violence for participants who worked in a hazardous occupation and who did not work in a hazardous occupation in the past year. We then specified GLMs with past-year hazardous work in young adulthood as the outcome and any experience of physical and/or sexual violence in childhood as the exposure. In the models for Kenya, given that the VACS included average hours of work per day, we examined working more than 9 h on average in a day as an additional outcome.

Analyses were conducted in Stata 16 and R. All analyses were country specific, stratified by sex, and survey weighted to account for the survey design. We report adjusted odds ratios (aORs) and 95% CIs for all regression models. Given that we conducted a number of comparisons and that findings could be significant by chance, we interpreted both the statistical significance and the pattern of the findings.

## 3. Results

### 3.1. Study Sample

We included data from 35,723 young people aged 13–24 years in nine countries from VACS surveys conducted between 2013 and 2018 (Table 1). Our sample includes LMICs in Sub-Saharan Africa (6 countries) and Latin America (3 countries). Sample sizes for young women ranged from 891 (Zambia, 2014/15) to 7912 (Zimbabwe, 2017), and for young men, they ranged from 803 (Zimbabwe) to 2659 (Honduras, 2017).

Prevalence of children’s and young people’s work:

Table 1 shows the sex-stratified prevalence of past-year work and work in a hazardous site among 13–24-year-olds in each country. Past-year work was highest in Malawi: 79.7% of girls/young women (95% CI: 74.5, 84.0) and 82.4% of boys/young men (95% CI: 78.6, 85.7) reported work. Past-year work was lowest in El Salvador: 26.1% (95% CI: 22.8, 29.5) of girls/young women and 43.9% of boys/young men (95% CI: 40.4, 47.6) reported past-year work.

Among the seven countries with comparable data on worksites, the prevalence of work in a hazardous site among girls/young women ranged from 1.0% (95% CI: 0.8, 1.4) in Zimbabwe to 3.9% (95% CI: 2.3, 6.6) in Malawi. Among boys/young men, work in a hazardous site ranged from 4.8% (95% CI: 3.9, 5.8) in Uganda to 18.2% in Honduras (95% CI 15.7, 21.0). Work in a hazardous site was higher among boys/young men compared to girls/young women in every country, and it was four times higher in In El Salvador, more than six times higher in Nigeria and Zambia, and more than eight times higher in Honduras and Zimbabwe. The Kenya VACS was the only survey where participants were asked specific questions about working in a factory/workshop, construction site, mine/quarry, over open flames, or with sharp tools or heavy equipment. The prevalence of hazardous work was highest among girls/young women (7.2%, 95% CI: 5.2, 9.8) and boys/young men in Kenya (33.9%, 95% CI: 30.0, 38.1), where one in three boys/young men reported at least one form of hazardous work.

**Figure 1:**
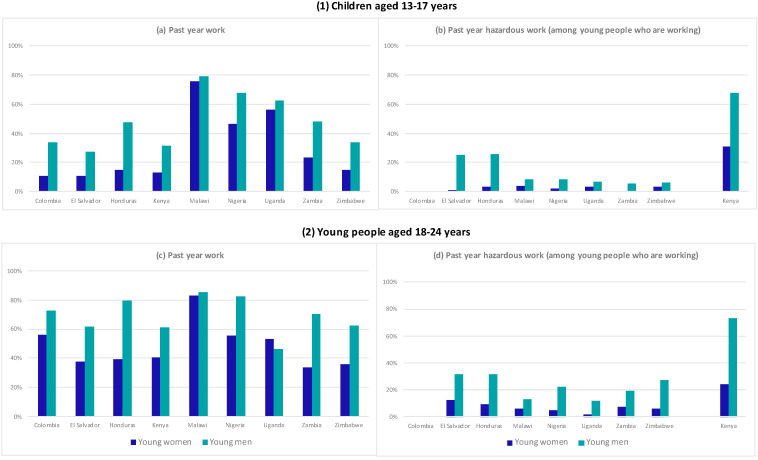
Sex- and age-stratified prevalence of (**a**) past-year work and (**b**) hazardous work among children aged 13–17 years who are working. Sex- and age-stratified prevalence of (**c**) past-year work and (**d**) hazardous work among young people aged 18–24 years who are working.

Figure 1 shows the prevalence of work stratified by age and sex (girls/boys 13–17 years compared to young women/men aged 18–24 years), and among those working, the proportion of work in a hazardous site (factory/workshop, construction site, or mine/quarry). In every country, among both age groups, the prevalence of work was higher among boys and young men compared to girls and young women. We describe results among children first before turning to young people.

Among girls aged 13–17 years, the prevalence of past-year work was highest in Malawi (75.6%, 95% CI: 68.1, 81.9) and Uganda (56.1%, 95% CI: 50.0, 62.0) and lowest in El Salvador (10.7%, 95% CI: 7.7, 14.7). Among boys aged 13–17 years, the prevalence of work was also highest in Malawi (78.9, 95% CI: 74.9, 82.5) and, additionally, in Nigeria (67.7%, 95% CI: 63.8, 71.4). The prevalence of work in a hazardous site was highest in Kenya for both girls (30.9%) and boys (67.5%). In the seven countries with comparable data, less than 5% of girls who were working reported work in hazardous sites (range: 1.2–3.6%). Among boys who were working, between 5.7% to 25.6% worked in hazardous sites. In addition to Kenya, in El Salvador and Honduras, more than 20% of boys’ work took place in hazardous sites.

Among young women aged 18–24 years, the prevalence of past-year work was highest in Malawi (82.8%, 95% CI: 77.7, 86.9), followed by Colombia (55.7%, 95% CI: 47.8, 63.4) and Nigeria (55.7%, 95% CI: 50.8, 60.4). Among young men, more than 60% reported work in every country except Uganda (46.1%, 95% CI: 42.5, 49.8). Past-year work was highest in Malawi (85.6%, 95% CI: 79.4, 90.1) and also above 70% in four other countries (Colombia, Honduras, Nigeria, and Zambia). The proportion of work that took place in a hazardous site was also highest in Kenya among both young women (24.1%) and young men (73.2%). Among young women who were working, work in hazardous sites was highest in El Salvador (11.9%, 95% CI: 8.16, 17.09) and less than 10% in all the other countries. Among young men, after Kenya, work in hazardous sites was highest in El Salvador and Honduras—where one in three young men worked in a hazardous worksite.

### 3.2. Locations of Young People’s Work

**Figure 2:**
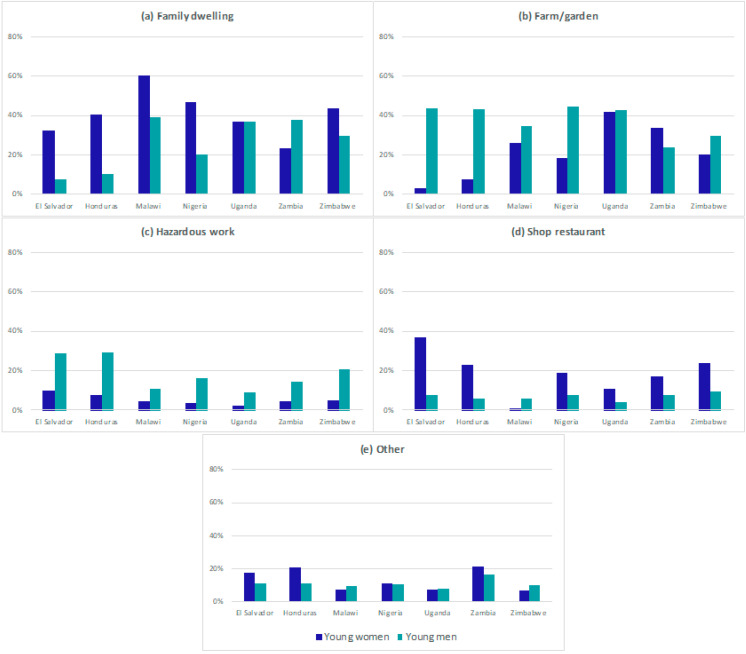
Worksites for past-year work among young women and men in 7 countries.

Among young people who worked in the past year, we examined data on worksites in seven out of nine countries (no comparable data in Kenya and Colombia). The location of young people’s work varied by country and by sex (Figure 2). Across the majority of countries, young women were more likely to be working in family or domestic dwellings (range: 23.5–60.6%) compared to men (range: 8.0–39.0%), while men were more likely to be working on a farm (range: 24–44%) compared to women (range: 3–42%). Zambia was the only country where more women reported farm work than men, and more men reported work in family dwellings compared to women. In six countries, women reported working in shops/restaurant more often than men. Across countries, between 7% and 21% of both men and women selected “other” for worksite. In six surveys, participants were asked to list their worksite if they selected “other”. Open text responses captured some additional worksites where other forms of work were performed, including “moulding bricks” (Malawi), “cattle” (Zimbabwe), or other forms of manual or physical labour among men. Among women, open text responses included “working in a school” and “saloon” (Uganda). We also found that some worksites may have been misclassified as “other”—for example, “maid” or “weeding” could have been coded as “family dwelling” or “farm/garden,” respectively Supplementary Figure S1 shows word clouds for the “other” category in each country. In the Kenya survey, the location of work was not prespecified, and women reported farming, business, housework, and selling as the most frequent work, while men reported farming, casual labour, and construction.

### 3.3. Decision-Making about Money Earned from Work

In the five countries with data available on decision-making, most boys/young men and girls/young women reported that they decided themselves how money earned would be spent: this ranged from 90.7% of girls/young women and 86.7% of boys/young men in Colombia to 61.0% of girls/young women and 62.0% of boys/young men in Uganda, with no notable differences in decision-making by sex. In most countries, parents were the second most common decision-maker—ranging from 26% of boys/young men in Uganda reporting parental involvement to 1% in Colombia. In most countries, less than 10% of participants reported that a partner decided how money was spent or that decision-making was shared with someone else. An exception was the Uganda VACS, where 11.5% of girls/young women reported that a partner decided how money was spent, while no boys/young men reported that their partners decided. Supplementary Table S2 presents country-specific worksite and decision-making results.

### 3.4. Associations between Past-Year Work and Violence among Children and Young People

**Figure 3:**
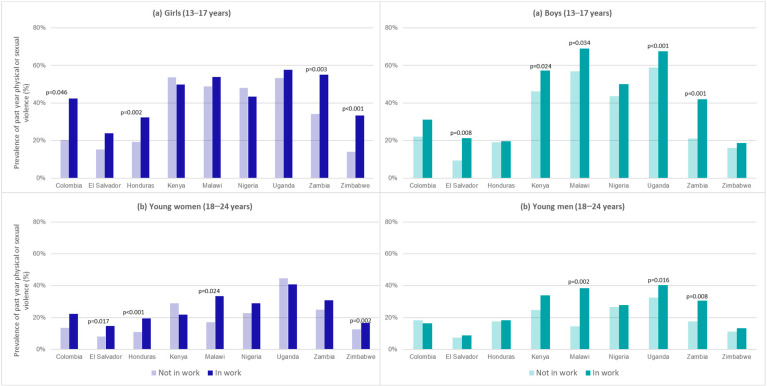
Prevalence of past-year physical and/or sexual violence by past-year work stratified by age and sex.

The second aim of this paper is concerned with associations between past-year work (exposure) and violence (outcome). Figure 3 presents the prevalence of any past-year violence among those who did and did not work in the past year for each country stratified by sex and age. Importantly, this is violence from any adult, partner, caregiver, or peer and may or may not include violence in the workplace. In the majority of countries, the prevalence of past-year physical and/or sexual violence was higher among children aged 13–17 years compared to 18–24 years and among young people who were working, although there is substantial variation both between countries and within country by sex and age. In the few cases where the prevalence of violence was lower among young people who were working, these differences were not statistically significant. Among children aged 13–17 years, the prevalence of past-year physical and/or sexual violence was statistically significant and higher among girls who were working compared to those not working in four countries (Colombia, Honduras, Zambia, and Zimbabwe) and among boys in four countries (El Salvador, Kenya, Malawi, and Zambia). Among young people aged 18–24 years, we found significant bivariate associations among young women in four countries (El Salvador, Honduras, Malawi, and Zimbabwe) and among young men in three countries (Malawi, Uganda, and Zambia).

**Figure 4:**
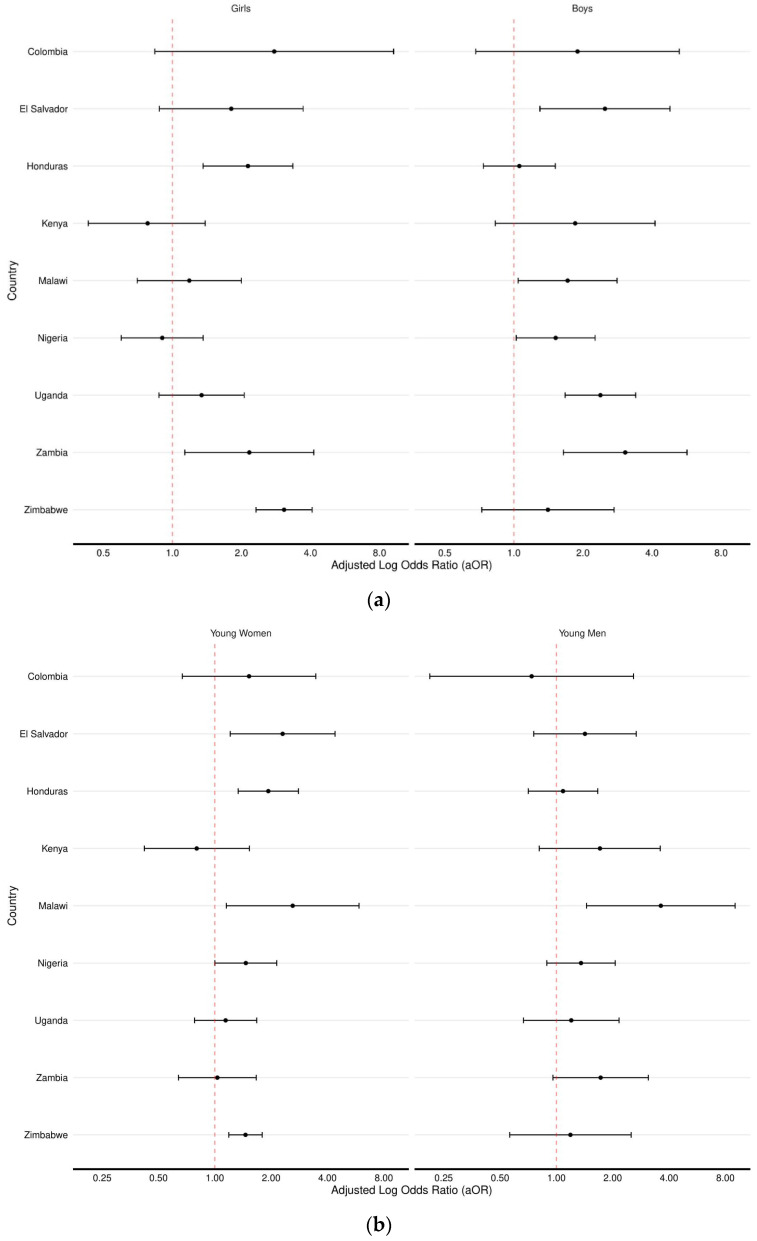
Associations between past-year work and violence stratified by sex among (**a**) children (13–17 years) and (**b**) young people (18–24 years). Reference group for the violence variables is no physical, sexual, or emotional violence in childhood. Covariates include age, parental death, ever married, type of toilet facility, sex of head of household, and ever attended school.

We then examined adjusted associations between past-year work and violence stratified by age and sex (Figure 4). Among children 13–17 years, the odds of past-year violence were higher among girls who were working compared to girls who were not working in three countries: aORs ranged from 2.14 in Honduras (95% CI: 1.36, 3.35) to 3.07 in Zimbabwe (95% CI: 2.32, 4.07). Among boys, aORs were significant in five countries, ranging from 1.46 in Uganda (95% CI: 1.13, 1.89) to 3.06 (95% CI: 1.64, 5.69) in Zambia.

Among young people aged 18–24 years, we found significant associations between past-year work and violence among young women in five countries: aORs ranged from 1.46 in Zimbabwe (95% CI: 1.19, 1.79) and Nigeria (95% CI: 1.00, 2.14) to 2.61 in Malawi (95% CI: 1.15, 5.90). Among young men, past-year work was associated with increased odds of violence in one country: in Malawi, boys who were working had 3.62 (95% CI: 1.45, 9.02) times higher odds of experiencing violence compared to boys who were not working. In the majority of countries where estimates were not significant, the direction and magnitude of estimates suggest increased odds of violence for children/young people engaged in work in the past year.

### 3.5. Associations between Childhood Violence and Work in a Hazardous Site among 18–24-Year-Olds

Our third and final aim was to examine whether childhood violence (exposure) was associated with working in a hazardous worksite in the past year (outcome). We restricted our sample to 18–24-year-olds so that childhood violence occurred before past-year work in a hazardous site. Supplementary Table S3 presents the prevalence of childhood violence by work in a hazardous worksite in the past year. Among young women, Nigeria was the only country with significant differences in later-in-life hazardous work outcomes by experiences of childhood violence: 84.6% of women aged 18–24 working in a hazardous site reported childhood physical and/or sexual violence compared to 63.2% of women who were not in a hazardous worksite (*p* = 0.019): both childhood sexual (*p* = 0.023) and physical violence (*p* = 0.011) were also higher among women working in a hazardous site. We found a higher prevalence of childhood physical and/or sexual violence among young men working in a hazardous site in Kenya (*p* < 0.001), Malawi (*p* = 0.027), and Uganda (*p* = 0.013), as well as in Honduras, among men who had experienced sexual violence (*p* = 0.019).

Table 2 shows adjusted odds ratios for associations between childhood physical and/or sexual violence and hazardous work in the past year. Among young women, we found significant positive associations in Nigeria, where the odds of working in a hazardous site were 3.21 (95% CI: 1.04, 9.93) times higher among women who had experienced childhood physical and/or sexual violence compared to those who had not experienced any violence in childhood. Among men, experiencing physical and/or sexual violence in childhood was associated with increased odds of being in a hazardous worksite in three countries—Kenya (aOR 5.21, 95% CI: 2.46, 11.02), Malawi (aOR 2.78, 95% CI: 1.21, 6.38), and Zimbabwe (aOR 2.54, 95% CI: 1.19, 5.42). In the countries where associations were not significant, the direction of association indicated an increased risk of hazardous work among young men.

Kenya was the only survey that asked young people about hours of work. We found significant associations between childhood violence and working more than 9 h a day on average. Young women who experienced physical and/or sexual violence in their childhood had three times higher odds of working an average of 9 h or more in a day (aOR: 2.82, 95% CI: 1.43, 5.57). Associations were not significant among young men.

## 4. Discussion

In this multi-country study on young people’s work and experiences of violence, we draw on data from over 35,000 children and young people aged 13–24 years across VACS in nine countries. We aimed to (1) describe the prevalence of children’s and young people’s work, (2) examine how the prevalence of physical and/or sexual violence varied by employment status, and (3) examine whether experiencing childhood violence was associated with adverse work outcomes in young adulthood.

We documented a high prevalence of work across countries with large variation by country, age, and sex, affirming the context-, gender-, and age-specific nature of work. Similar to other studies [1,43], we found that boys and young men are more likely to be in work and be working in a hazardous site compared to girls and young women. Although the prevalence of past-year work was higher among young people aged 18–24 years compared to children aged 13–17 years, we also show that a high proportion of children below 18 years were engaged in work: over 50% of boys were working in Nigeria, Malawi, and Uganda, and over 50% of girls were working in Malawi and Uganda. However, it is challenging to discern whether this constitutes child labour since the measures of past-year work used in the VACS does not take into account hours worked or working conditions. However, we also showed that, in three countries (Kenya, El Salvador, and Honduras), at least one in four boys were working were in a hazardous site—factory/workshop, construction site, or mine/quarry among boys—which meets global definitions of child labour and hazardous work [2]. For older children, although our findings reveal high engagement in work and, in some countries, in a hazardous worksite, beyond this, given the lack of information about the hours, seasonality, type of work, and wages, it becomes challenging to comment on experiences of work, access to the labour market, wage equity, or unemployment based on these prevalence estimates.

We also presented a range of exploratory analyses to examine links between work and violence and between childhood violence and past-year work. Our findings show that children and young people who work generally report a higher prevalence of past-year physical and/or sexual violence from any perpetrator. After adjusting for covariates, we documented significant positive associations between past-year work and violence which vary by both age and sex: among 13–17-year-old girls in three countries and boys in five countries, and among 18–24-year-old women in five countries and men in one country. Importantly, findings are not significant in some countries because the prevalence of past-year violence is high for both boys and girls in the VACS overall, irrespective of work status [44]. Taken together, these findings underscore the importance of considering age and gender when exploring links between work and violence and of workplaces as key sites for violence prevention [19,20,22,23,24].

Finally, we find that childhood violence is associated with work in a hazardous worksite in early adulthood among young women (Nigeria) and among young men (Kenya, Malawi, and Zimbabwe), and also with working long hours among women in Kenya. The theory of Developmental Traumatology outlines how chronic trauma in childhood and adolescence may affect the developing brain, influencing an individuals’ behavioural, cognitive, and emotional development, and ultimately causing a range of functional impairments, including adverse vocational and work outcomes [45,46]. Although there are a limited number of works in the literature on the relationship between childhood violence and exposure to hazardous worksites later in life, several other studies show links between childhood violence and other adverse work outcomes. A study using cross-sectional data from the Egypt Demographic and Health Survey among children aged 5–17 years found that experiencing violence (physical and severe punishment) was associated with hazardous labour [47]. Longitudinal studies in South Africa, the US, the UK, Denmark, and Norway find that child maltreatment increases the risk of adverse work outcomes [29,30,48,49,50]. A systematic review of longitudinal research on child maltreatment and later work outcomes (reduced income, unemployment, lower level of job skill, and fewer assets) further underscores that that childhood experiences of maltreatment increased the risk of adverse work outcomes after accounting for family of origin socio-economic status [27]. Our findings draw attention to how associations differ by sex and suggest hypotheses about the links between childhood violence and later-in-life work outcomes should be further explored in cross-sectional data where violence occurs prior to work outcomes, or in longitudinal data.

This study has both strengths and limitations. We draw on nationally representative data with large sample sizes, which are comparable across countries to generate some of the first multi-country estimates of violence by employment status and to describe the epidemiology of work. Additionally, we are able to present country-specific and sex- and age-stratified findings, generating new evidence about links between work and violence at different points of the life course.

The first set of limitations concerns the measurement of work and violence in the VACS. The definition of work may vary both within and across countries; there are no measures of hours, wages, or seasonality of work. This means that young people with heterogenous work experiences (e.g., engaging in seasonal work, temporary work, or daily work) are all coded as working. The measurement of work has implications on the prevalence estimates we report. For example, in the Kenya VACS, participants were asked about specific forms of hazardous work, resulting in a higher prevalence of hazardous work than any of the surveys where hazardous worksites were grouped together and included as one of the response options for location of work. Furthermore, we show that some worksite categories may be misclassified, and there may be additional meaningful categories. Given that employer violence is not specifically measured or asked about in the VACS, and employers are included in a list of perpetrators, we are unable to estimate if or how often children and young people experience employer or workplace violence. Other authors who have used VACS to examine who perpetrates violence against children are also unable to include employers or worksites in their analyses [44,51]. Although all surveys measure the same violence constructs, there are also differences between surveys in the items used to measure physical and emotional violence. It is likely that we underestimate both the prevalence of violence and of work and that the underestimation may be related to age and worksite. For example, an analysis of the VACS data from Haiti found that child domestic servants may have been under-sampled if the head of household did not include them in the household listing, and there were high rates of refusal by guardians to allow children to complete the survey [23]. Furthermore, as household surveys, the VACS are unlikely to include children who are engaged in the most hazardous forms of labour who live in their worksites, who are not attached to a household, or who are street connected.

A second limitation is related to our use of cross-sectional surveys. Although we examined whether past-year work is associated with violence, associations could be bidirectional. Although we restricted the sample to young people 18–24 years in our analyses of childhood violence and work in hazardous sites, and childhood violence is measured prior to the work outcome, childhood violence may continue into adolescence and adulthood and may still have occurred in the same year as the work for young people close to 18 years of age. Our approach to modelling work and violence also has limitations. We included physical and/or sexual violence as binary variables and did not examine the severity or perpetrators. Our “exposed” group is therefore likely to be very heterogeneous. The violence variables we used consisted of numerators where participates reported physical or sexual violence, and the denominators were young people who did not report physical, sexual, or emotional violence in order to create a comparison group that was “no violence”: therefore, young people who experienced emotional violence but not physical or sexual are not included in the numerator or denominator. Although we adjust our models for theoretically relevant covariates, there is likely to be residual and unmeasured confounding, as there are covariates that we were unable to include (including measures of agency, empowerment, and income) and limits to our measures of socioeconomic status (type of toilet facility and ever attending school). Given the number of comparisons and tests we report, our results should be interpreted as patterns overall rather than through focusing on particular associations which may have arisen by chance, and several of our estimates have large confidence intervals that indicate low precision and should be seen as hypothesis generating.

Our findings have several implications for further research on young people’s work and violence, as well as for violence prevention interventions and policies. First, our study underscores the importance of VACS in revealing the prevalence of work and violence in many LMICs [34]. We show that the VACS data can be used to construct nationally representative estimates of past-year work and the location of work, including in a hazardous site. Both are important, as we find that measuring only work could obscure the proportion of young people in hazardous work. For example, among boys, El Salvador had the lowest prevalence of work, but it is a country with one of the highest proportions of boys working in hazardous sites. Beyond the prevalence of work, VACS also allow researchers to begin to understand where young people are working: as we show, young women in most countries were working within the home or in shops/restaurant more than men, while young men worked in a farm/garden or a hazardous site more than women. Finally, VACS allows decision-making about money earned from work to be explored, and we show that, in most countries, young people decided themselves how their money would be spent, followed by parents or partners as other primary decision-makers. These data offer researchers and policy makers important opportunities to allow data to inform efforts to monitor, prevent, and respond to hazardous work, violence, and access to the labour market for young people. For example, the estimates we present could serve as baseline estimates for future surveys and could further reveal how work varies by gender, age, geography, and other factors over time.

Continuing to measure children’s and young people’s work in large-scale surveys is essential, as is improving the measurement of work and violence. Other research also points to the challenges of measuring children’s work, working hours, and worksites, as children’s work is often seasonal or sporadic, consists of multiple jobs, and occurs in the informal sector [23,52,53,54]. VACS could further refine the work questions to include a measure of the number of hours worked, ask if young people hold multiple jobs, explicitly measure employer violence and other violence that occurs at work (e.g., from customers or co-workers), and measure help seeking following workplace violence. This is particularly important since most young people work in the informal sector, which is not subject to the same regulation. Further efforts are also needed to improve the measurement of worksites and work types and to explicitly ask about other labour and work activities that young people engage in, including unpaid work and work at home. Such measurement decisions will have important implications on the prevalence of work and hazardous work. For example, fewer women reporting work compared to men is likely related to challenges with measuring women’s work. Efforts to improve measurement should also be paired with efforts to improve the reporting of work. For example, laws, norms, and stigma about the acceptability of work or what constitutes work could all affect how a participant reports work in an interview. Finally, our findings also raise questions about the measurement of both the locations of young people’s work and the type of work. The write-in data in the “other” category indicate that there may be misclassification and measurement error in the measures of worksites. For example, although “office work” and “housework” were response options, they often appeared under “other”. Such misclassification may lead to an underestimation of how many young people engage in a particular type of work. Worksites that were included in the “other” category could be used to improve response options in future efforts to measure worksites.

Our findings also have implications for research and could be used to generate hypotheses for further research on children’s work and violence. First, new surveys on work and violence conducted with young people should include questions about workplace violence and consider the many types of permanent, temporary, or seasonal work young people participate in. Data on workplace violence come mainly from studies of specific sectors, for example, agriculture, mining, and domestic work [55,56,57], rather than across sectors; however, a study in Uganda found that workplace violence among young people was common across sectors, with two in five reporting workplace violence in the past year [21]. This makes it challenging to monitor and prevent workplace violence, and future research should be designed to inform prevention efforts. Secondly, further country- and context-specific research is required to better understand the links between work and violence. Intersectional analyses are key here, and such research should continue to pay attention to gender and age and could additionally examine how work outcomes vary by disability, poverty, and rurality. Future research that uses cohort or longitudinal data where the violence exposure was assessed prior to the work exposures is crucial in examining the pathways between childhood violence and adverse work outcomes (e.g., hazardous work, long hours, pay gaps, and workplace violence) to inform opportunities for prevention, and such studies are particularly needed in LMICs, since data are almost exclusively from high-income countries. Our analysis did not examine violence by perpetrators; including this in future analyses could further inform prevention efforts and better explain how being in work changes who is perpetrating violence against young people.

The implications for policy and practice relate to the continued need to prevent violence and improve working environments for young people, regulate and prevent child labour [58], and consider how to integrate interventions for violence prevention and response within workplaces. Given the high prevalence of both violence and work that we are reporting, as well as the higher prevalence of violence among those working, workplaces should be central to a commitment to violence prevention that is multi-sectoral and works across the social ecology. Engaging workplaces in violence prevention and addressing the barriers—including violence—that could affect access to safe and paid work are both important [59]. Country- and context-specific efforts are essential and could focus on forms of violence that occur outside the workplace, as well as workplace violence, and should include the informal sector where many young people work. Although we could not include workplace violence in this study, global activism and campaigns, including the #MeToo and #TimesUp movements [60,61], have drawn attention to the widespread nature of workplace violence emotional, sexual, and physical violence. UN Conventions, including the ILO 2019 Convention to prevent and eliminate violence and harassment in the workplace, and national policies have also endeavoured to change policies to prevent violence in the workplace. Importantly, the home is also a site of workplace violence for many young women who work as domestic workers [55], underscoring the importance of continued efforts to prevent violence at the household and community level and consider these contexts as workplaces.

## 5. Conclusions

We documented a high prevalence of past-year work across nine countries and showed that, in many countries, violence is higher among children who are working. We find positive associations between past-year violence and work, as well as between childhood violence and hazardous work. Our findings draw attention to workplaces as sites of violence prevention, as well as the importance of preventing violence, adverse work outcomes, and their intersections. The fact that both experiencing violence and being in hazardous work in childhood are associated with adverse health, education, economic, and social outcomes [31,62,63,64,65] highlights the need to think concurrently about work and violence for children and young people both within and beyond workplaces.

## Figures and Tables

**Table 1:** Prevalence of past-year and hazardous work in the Violence Against Children Surveys in 9 countries.

Country	Young Women (13–24 Years)									Young Men (13–24 Years)								
	Sample Size	In Work (Past Year; Paid or Unpaid)				Hazardous Work (Past Year)				Sample Size	In Work (Past Year; Paid or Unpaid)				Hazardous Work (Past Year)			
		n	%	95% CI		n	%	95% CI			n	%	95% CI		N	%	95% CI	
Colombia, 2018	1406	499	37.0%	31.7	42.6	Not comparable				1299	647	56.4%	49.7	63.1	Not comparable			
El Salvador, 2017	1056	268	26.1%	22.8	29.5	33	2.6%	1.7	4.0	1380	608	43.9%	40.4	47.6	178	12.7%	10.5	15.4
Honduras, 2017	2537	723	28.5%	26.4	30.7	51	2.2%	1.6	3.0	2659	1596	63.2%	60.3	66.1	476	18.2%	15.7	21.0
Kenya, 2010 *	1227	362	28.1%	24.1	32.1	103	7.2%	5.2	9.8	1456	671	47.5%	43.2	51.7	493	33.9%	30.0	38.1
Malawi, 2013	1029	784	79.7%	74.5	84	39	3.9%	2.3	6.6	1133	924	82.4%	78.6	85.7	115	9.0%	7.3	11.1
Nigeria, 2014	1766	907	51.9%	47.4	56.4	45	2.0%	1.3	3.0	2437	1823	75.9%	73.1	78.5	315	12.6%	10.6	15.0
Uganda, 2015	3159	1728	54.2%	49.9	58.4	32	1.4%	0.6	3.1	2645	1482	54.6%	52.1	57.1	126	4.8%	3.9	5.8
Zambia, 2014/2015	891	261	28.8%	25.7	32.1	11	1.3%	0.7	2.5	928	556	60.4%	56.4	64.3	82	8.5%	6.2	11.5
Zimbabwe, 2017	7912	2081	26.7%	25.4	28	78	1.0%	0.8	1.4	803	396	48.8%	44.6	52.9	68	8.6%	6.3	11.7

Notes: Percentages, n, and 95% CIs are survey weighted. Sample sizes represent the unweighted sample. In all countries except Kenya, hazardous work includes working in a factory/workshop, construction site, or mine/quarry. * In Kenya, hazardous work includes working in a mine; over open flames; with sharp tools, such as a machete; with heavy equipment; or in a factory.

**Table 2:** Associations between childhood violence and hazardous work in the past year among young women and men aged 18–24 years.

Country	Year	Young Women					Young Men				
		aOR	95% CI		*p*	n	aOR	95% CI		*p*	n
		Hazardous Worksite									
Colombia	2018	N/A					N/A				
El Salvador	2017	1.35	0.47	3.88	0.575	568	2.15	0.84	5.51	0.112	637
Honduras	2017	0.74	0.37	1.47	0.389	1359	1.07	0.77	1.48	0.679	1180
Kenya *	2010	1.02	0.75	1.39	0.880	649	5.21	2.46	11.02	*p* < 0.001	318
Malawi	2013	1.61	0.53	4.90	0.397	492	2.78	1.21	6.38	0.016	438
Nigeria	2014	3.21	1.04	9.93	0.043	883	1.40	0.94	2.10	0.098	1215
Uganda	2016	4.08	0.97	17.08	0.055	1541	1.30	0.70	2.42	0.400	1126
Zambia	2014/2015	0.63	0.09	4.44	0.637	413	1.27	0.67	2.44	0.461	441
Zimbabwe	2017	0.60	0.30	1.19	0.143	4283	2.54	1.19	5.42	0.017	388
		**Over 9 h worked**									
Kenya	2010	2.82	1.43	5.57	0.004	607	0.95	0.40	2.25	0.905	313

Notes: Sample restricted to 18–24-year-olds. Reference group for the violence variables is no physical, sexual, or emotional violence in childhood. Covariates include age, parental death, child marriage, type of toilet facility, and sex of head of household. In all countries except Kenya, hazardous work includes working in a factory/workshop, construction site, and mine/quarry. * In Kenya, hazardous work includes working in a mine; over open flames; with sharp tools, such as a machete; with heavy equipment; and in a factory.

## Data Availability

VACS data are publicly available on the Together for Girls website (https://www.togetherforgirls.org/violence-children-surveys).

## Supplementary Materials

The following supporting information can be downloaded at https://www.mdpi.com/article/10.3390/ijerph192416936/s1. Table S1: Measures of work in the Violence Against Children Surveys (VACS). Table S2: Worksites and decision-making about money among young people aged 13–24 years. Figure S1: Open text responses word for worksites by sex and country. Table S3: Childhood violence and work in a hazardous worksite in the past year among young people aged 18–24 years.
