# Supplementary material for: The Epidemiology of Young People’s Work and Experiences of Violence in Nine Countries: Evidence from the Violence against Children Surveys
Source: Int J Environ Res Public Health. 2022 Dec 16;19(24):16936. doi: 10.3390/ijerph192416936 (PMC9778926; doi:10.3390/ijerph192416936)
Supplement: Supplementary file 1 [file ijerph-19-16936-s001.zip › ijerph-1995051-supplementary.pdf]

Supplementary Table S1: Measures of work in the Violence Against Children Surveys (VACS)

<table>
<tr><th rowspan="2">Work Variable</th><th colspan="9">Country</th></tr>
<tr><th>Colombia</th><th>El Salvador</th><th>Honduras</th><th>Kenya</th><th>Malawi</th><th>Nigeria</th><th>Zambia</th><th>Zimbabwe</th><th>Uganda</th></tr>
<tr><td>Past year work</td><td colspan="3">In the last 12 months did you engage in any work as an employee, or self-employed individual?

1=Yes
2=No
98=Don't know
99=Declined</td><td>In the last 12 months, how often have you worked for money or any other payment/goods?

1=Throughout the year
2=Seasonally/Part of the year
3=Once in a while
4=Not at all
99=No response</td><td colspan="5">At any time during the past 12 months did you engage in any work?

1=Yes
2=No
99=Don't know/declined</td></tr>
<tr><td>Worksite</td><td>Data not comparable</td><td colspan="2">Where did you carry out your main work during the past 12 months?

1=At family dwelling (domestic work/foods for sale/handicraft products)
2=Formal office
3=Factory/workshop
4=Farm/garden
5=Construction site
6=Mine/quarry
7=Shop/kiosk
8=Restaurant/hotel/café, bar
9=Different places (mobile)
10=Fixed, street or market stall
11=Pond/lake/river
88=Other (specify)
98=Don't know
99=Declined</td><td>In the last 12 months when you were working for money/goods did you work in a mine, work over open flames, work with sharp tools such as a machete, work with heavy equipment, or work in a factory?
1=Yes
2=No
3=No response</td><td colspan="5">Where did you carry out your main work during the past 12 months?

1=At family dwelling
2=Formal office
3=Factory/workshop
4=Farm/Garden
5=Construction site
6=Mine/quarry
7=Shop/kiosk
8=Restaurant/hotel/café, bar
9=Different places (mobile)
10=Fixed, street or market stall
11=Pond/lake/river
88=Other (specify)
98=Don’t know
99=Declined</td></tr>
</table>

Work Variable	Country								
	Colombia	El Salvador	Honduras	Kenya	Malawi	Nigeria	Zambia	Zimbabwe	Uganda
Decision-making	Who usually decides how the money you earn will be used?<br><br>1=I decide<br>2=I decide jointly with someone<br>3=My husband decides<br>4=My boyfriend decides<br>5=My parents decide<br>6=Someone else decides<br>98=Don't know<br>99=Declined	Who usually decides how the money you earn will be used?<br><br>1=I decide<br>2=My husband decides<br>3=My boyfriend decides<br>4=My parents decides<br>5=Someone else decides<br>6=I decide jointly with someone<br>98=Don't know<br>99=Declined		In the past 12 months, when you were working for money or other payment, who mainly decides how to spend the money you earn?<br><br>1=Respondent<br>2=Husband/Partner<br>3=Parent decide<br>4=Respondent and someone else jointly<br>5=Other<br>99=No response	Not asked			In the past 12 months, when you were working for money or other payment, who mainly decided how to spend the money you earned?<br><br>1=I decide<br>2=My husband decides<br>3=My boyfriend decides<br>4=My parents decides<br>5=Someone else decides<br>6=I decide jointly with someone<br>98=Don't know	Who usually decides how the money you earn will be used?<br><br>1=I decide<br>2=My husband decides<br>3=My boyfriend decides<br>4=My parents decides<br>5=Someone else decides<br>6=I decide jointly with someone<br>98=Don't know

Supplementary Table S2: Worksites and decision making about money among young people aged 13-24 years

Country	Year	Sample size	Worksites (past year)										Decision making about money earned from work									
			Family dwelling		Farm/ garden		Shop/ restaurant		Hazardous work site		Other		I decide		Joint decision		Partner decides		Parent decides		Someone else decides	
			n	%	n	%	n	%	n	%	n	%	n	%	n	%	n	%	n	%	n	%
**Female**																						
Colombia	2018	1406	Not comparable										418	90.7%	42	7.8%	9	0.7%	8	0.8%	2	0
El Salvador	2017	1056	88	32.3%	9	3.3%	93	36.9%	33	10.1%	45	17.4%	189	75.8%	21	10.8%	4	0.8%	25	11.3%	3	1.3%
Honduras	2017	2537	285	40.5%	56	7.9%	176	23.1%	51	7.6%	154	20.9%	563	83.3%	41	5.5%	8	1.1%	57	8.7%	5	1.4%
Kenya	2010	1227	Not measured										221	63%	32	9.0%	35	8%	63	17.0%	10	3.0%
Malawi	2013	1029	484	60.6%	183	26.1%	16	1.1%	39	4.9%	61	7.3%	Not measured									
Nigeria	2014	1766	411	47.1%	164	18.6%	180	19.3%	45	3.8%	107	11.2%	Not measured									
Uganda	2015	3,159	582	36.9%	794	41.5%	198	11.3%	32	2.6%	121	7.7%	295	61.0%	60	6.2%	59	11.5%	92	19.0%	18	2.3%
Zambia	2014/2015	891	57	23.5%	96	33.7%	45	17.0%	11	4.5%	51	21.3%	Not measured									
Zimbabwe	2017	7912	662	43.7%	316	20.3%	372	23.9%	78	5.2%	116	6.9%	570	78.8%	33	4.8%	16	2.5%	77	9.9%	28	4.0%
**Male**																						
Colombia	2018	1299	Not comparable										526	86.7%	46	6.7%	7	0.4%	35	6.2%	0	0.0%
El Salvador	2017	1380	49	8.3%	250	43.6%	55	7.9%	178	29.2%	69	11.1%	414	78.0%	34	5.4%	8	1.9%	68	13.6%	5	1.1%
Honduras	2017	2659	166	10.6%	633	43.1%	101	5.9%	476	29.1%	204	11.3%	1101	78.5%	92	6.3%	21	1.3%	189	13.7%	4	0.3%
Kenya	2010	1456	Not measured										476	71.0%	24	3.0%	5	0.4%	148	23.0%	17	3.0%
Malawi	2013	1133	387	39.1%	274	34.7%	59	5.9%	115	11.0%	88	9.4%	Not measured									
Nigeria	2014	2437	359	20.5%	807	44.2%	156	7.9%	315	16.7%	186	10.8%	Not measured									
Uganda	2015	2,645	500	36.7%	690	42.7%	50	3.9%	126	8.7%	114	8.0%	297	62.0%	43	8.0%	2	0.3%	129	26.1%	18	3.4%
Zambia	2014/2015	928	198	37.7%	130	24.1%	46	7.6%	82	14.1%	99	16.5%	Not measured									
Zimbabwe	2017	803	107	29.8%	99	29.8%	34	9.7%	68	20.8%	31	10.0%	80	67%	7	4.9%	3	2%	22	15%	11	10%

Notes:

Percentages, n, and 95% CIs are survey weighted. Sample sizes represents the unweighted sample.

In all countries except Kenya, hazardous work includes working in a factory/workshop, construction site, mine/quarry. In Kenya hazardous work includes working in a mine, over open flames, with sharp tools such as a machete, with heavy equipment, in a factory

Shop/restaurant includes: working in a shop/kiosk or a restaurant/hotel/café/bar or in a fix, street or market stall

Decision making about money earned from work: In the Kenya VACS participants could not select "someone else decides", but could select "other"

Supplementary Figure S1: Open text responses word for work sites by sex and country

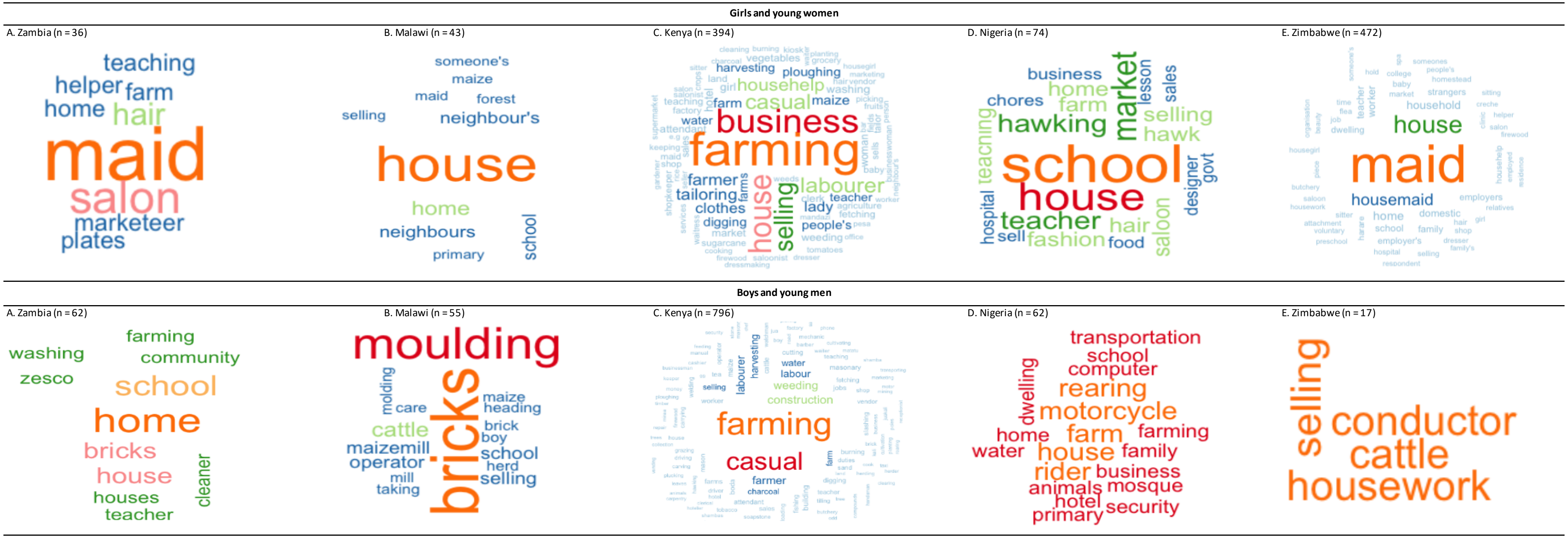

Notes:
No stemming or word correction has been done. Number of observations reflect number of respondents answering survey question, not number of word responses, Word frequencies >= 2 are shown. R Brewer Paired color palette is used (diverging color palette, set at 8 colours)
Most frequent words are shown as the largest and are in orange, second most frequent are in red, least frewquent words are the smallest and in light blue. No open text responses in El Salvador, Honduras, Colombia

Supplementary Table S3: Childhood violence and work in a hazardous worksite in the past year among young people aged 18-24 years

Violence in childhood	Young women (18-24 years)						Young men (18-24 years)					
	No work in hazardous worksite in the past year		Work in a hazardous worksite in the past year		*p*	N	No work in hazardous worksite in the past year		Work in a hazardous worksite in the past year		*p*	N
	%	SE	%	SE			%	SE	%	SE		
**Colombia**												
Physical or sexual violence	29.9%	4.2	26.0%	4.24	0.594	381	29.2%	3.37	25.4%	4.94	0.565	350
Sexual violence	19.1%	3.4	17.9%	3.42	0.805	232	9.7%	2.21	13.3%	4.42	0.474	98
Physical violence	22.3%	4.02	13.7%	3.9	0.147	233	25.5%	3.2	19.8%	4.9	0.360	291
**El Salvador**												
Physical or sexual violence	20.5%	1.77	20.7%	8.28	0.981	1011	14.4%	1.34	20.4%	5.97	0.292	1355
Sexual violence	12.9%	1.71	20.7%	8.28	0.258	922	3.5%	0.61	5.3%	2.15	0.341	1198
Physical violence	13.1%	1.33	3.0%	3.06	0.106	931	12.5%	1.32	17.5%	6.21	0.390	1322
**Honduras**												
Physical or sexual violence	41.3%	1.42	40.4%	8.08	0.911	2469	35.5%	1.35	41.7%	2.72	0.042	2621
Sexual violence	20.5%	1.18	23.3%	7.15	0.688	1825	11.6%	0.94	17.0%	2.17	**0.019**	1885
Physical violence	37.7%	1.47	35.8%	8.5	0.825	2,324	33.8%	1.36	39.6%	2.79	0.062	2538
**Kenya**												
Physical or sexual violence	78.1%	2.65	79.1%	5.74	0.864	1190	76.1%	2.12	89.5%	1.84	**<0.001**	1150
Sexual violence	55.9%	4.54	65.9%	8.08	0.264	579	33.0%	3.96	67.1%	5.16	**<0.001**	199
Physical violence	77.0%	2.82	76.6%	6.44	0.946	1132	75.8%	2.14	89.3%	1.88	**<0.001**	1128
**Malawi**												
Physical or sexual violence	62.1%	2.9	72.8%	9.36	0.370	969	76.8%	2.24	88.0%	3.56	**0.027**	1102
Sexual violence	42.6%	3.12	58.6%	9.00	0.143	584	40.0%	4.26	67.9%	9.35	**0.013**	400
Physical violence	59.6%	3.35	67.4%	14.4	0.648	908	76.4%	2.27	87.7%	3.65	**0.029**	1086
**Nigeria**												
Physical or sexual violence	63.2%	2.01	84.6%	6.49	**0.019**	1706	62.4%	1.76	68.5%	3.38	0.096	2318
Sexual violence	41.6%	2.45	72.5%	11.76	**0.023**	1057	24.6%	1.67	32.7%	4.48	0.069	1164
Physical violence	60.0%	2.16	84.0%	6.67	**0.011**	1584	61.2%	1.82	66.9%	3.56	0.132	2245
**Uganda**												
Physical or sexual violence	74.6%	1.76	58.7%	25.29	0.471	3011	78.8%	1.10	88.0%	2.87	**0.013**	2550
Sexual violence	58.8%	2.48	35.8%	25.49	0.382	1805	45.1%	2.11	67.8%	7.62	**0.006**	991
Physical violence	72.2%	1.93	54.1%	26.42	0.441	2811	78.3%	1.11	87.9%	2.88	**0.010**	2493
**Zambia**												
Physical or sexual violence	53.9%	2.21	35.5%	17.19	0.307	846	52.6%	1.92	53.9%	7.15	0.865	868
Sexual violence	34.3%	2.19	10.7%	10.55	0.150	606	17.1%	1.72	30.9%	8.22	**0.052**	518
Physical violence	49.0%	2.43	35.5%	17.19	0.457	760	50.8%	1.97	50.0%	7.4	0.919	827
**Zimbabwe**												
Physical or sexual violence	24.9%	0.75	21.2%	5.07	0.496	1,900	25.4%	2.99	37.3%	6.89	0.058	196
Sexual violence	8.3%	0.39	4.7%	3.55	0.443	515	0.7%	0.48	1.9%	1.89	0.371	4
Physical violence	21.7%	0.76	18.0%	4.69	0.459	1,594	25.4%	2.99	36.5%	6.91	0.077	195
